# Preliminary lessons from the GLENCOE farmlets: early trajectories of intensified grazing management on basaltic native grasslands

**DOI:** 10.1093/af/vfag025

**Published:** 2026-06-09

**Authors:** Thais Devincenzi, Martin Jaurena, Virginia Porcile, Fabio Montossi

**Affiliations:** Instituto Nacional de Investigación Agropecuaria (INIA), Estación Experimental INIA Tacuarembó, Tacuarembó, 45000, Uruguay; Instituto Nacional de Investigación Agropecuaria (INIA), Estación Experimental INIA Tacuarembó, Tacuarembó, 45000, Uruguay; Instituto Nacional de Investigación Agropecuaria (INIA), Estación Experimental INIA Tacuarembó, Tacuarembó, 45000, Uruguay

**Keywords:** Campos biome, cow–calf operations, long-term experiments, mixed grazing

Implications

- This study focuses on identifying the long-term trajectories generated by adaptive management in three grazing systems with increasing numbers of paddocks in the natural grasslands of northern Uruguay.
- Trajectories and performances are assessed through a multidimensional framework capturing productive, environmental, economic, human, and emergent properties.
- Increasing grazing management intensity, achieved by subdividing the grazing area into a greater number of paddocks, significantly influences early productivity and pasture dynamics. An innovative co-learning environment involving producers, extensionists, and researchers has been established during the first 3 yr of the study.

## Introduction

Native grasslands of the South American Campos biome support livestock production systems that are inherently high in species diversity and complex due to variations in plant communities in space and over time, driven by climatic fluctuations. These ecosystems extend across central-eastern Argentina, Uruguay, and southern Brazil and have underpinned extensive grazing livestock systems for more than three centuries. They are sustained by year-round grazing of highly biodiverse grasslands, which provide multiple ecosystem services (Jaurena et al., 2021). Moreover, the natural grasslands of this region represent a multifaceted production–conservation landscape, where the interplay between ecological persistence and livestock productivity reflects long-standing co-evolutionary dynamics and contemporary management challenges (De Faccio Carvalho and Batello, 2009).

Campos grassland is the sole biome in Uruguay and constitutes the ecological foundation of the country’s livestock-based economy and culture. Livestock production has historically relied on native grasslands, which have been extensively grazed since the introduction of cattle over 300 yr ago. Their persistence and productivity are the result of centuries of co-evolution between plant communities, grazing animals, and climatic variability. However, in recent decades, land-use change—including the conversion of native grasslands to cultivated pastures, annual crops, and forestry—has emerged as a major driver of biodiversity loss and degradation of ecosystem services (Baeza and Paruelo, 2020).

In extensive livestock systems based on native grasslands, mismatches between forage growth and animal demand remain a central constraint to productivity, economic performance, and ecosystem integrity. These mismatches are frequently amplified under increasing climatic variability and market uncertainty, leading to recurrent episodes of overgrazing, underutilization of forage, loss of plant diversity, and reduced system resilience (Briske et al., 2015; Jaurena et al., 2021). While substantial progress has been made in understanding grazing management at the paddock or system scale, there is still limited empirical evidence on how alternative grazing decisions applied consistently over time, shape long-term trajectories of productivity, stability, and ecosystem services at the scale of real production systems.

Addressing these knowledge gaps requires long-term experimental platforms explicitly designed to operate at the system level, where management decisions, biological responses, and feedback co-evolve through time. Long-term experiments allow the evaluation of cumulative and lagged effects that cannot be detected in short-term studies, particularly those related to resilience, stability, trade-offs among ecosystem services, and the adaptive capacity of grazing systems under climate uncertainty. Moreover, they provide a robust basis for developing decision-support frameworks grounded in observed system behavior rather than isolated responses.

The GLENCOE long-term experimental platform was conceived to respond to this challenge by evaluating contrasting levels of spatial and temporal grazing control in native grasslands under realistic livestock systems. The platform focuses on identifying trajectories generated by adaptive management, where stocking rate (SR) and grazing decisions are adjusted according to pre-established criteria in different paddock subdivision systems. In doing so, GLENCOE seeks to generate scientific evidence relevant not only to Uruguay but also to subtropical grassland regions, which face the dual challenge of increasing livestock productivity while maintaining ecosystem integrity.

Co-innovation has been conceptualized from multiple perspectives, with several authors emphasizing its role in enabling adaptive management in complex systems through the active involvement of stakeholders (Klerkx et al., 2010; Lee et al., 2012; Small et al., 2021). This co-innovation process highlighted the principles of collaboration, organization, complementarity, and co-creation with stakeholders (Lee et al., 2012). In the context of long-term experiments in a complex system, a support group of farmers, technicians, and researchers plays a key role in systemic thinking to analyze the experimental results, discuss adaptive management decisions, and facilitate the communication of technical messages to the farmer as detailed by Moojen et al. (2024).

Through GLENCOE Platform, we aim to 1) identify the long-term impacts of increasing levels of grazing management intensity on plant and animal production, and overall sustainability in native grasslands; 2) establish a support group to the long-term experiment integrated by the main stakeholders of livestock production in Uruguay to co-innovate in adaptive management decisions and to communicate knowledge.

We hypothesized that finer spatial and temporal control of grazing through increasing paddock subdivision enhances animal production, ecosystem services, and the resilience of grazing systems based on native grasslands.

In this paper, we present early insights from the first 3 yr of evaluation into the trajectories emerging from the three farmlets of the GLENCOE Platform, focusing on how contrasting levels of grazing management shape system responses over time.

## Experimental Design, Production Systems, and Key Variables Evaluated

### Platform design and experimental logic

The GLENCOE long-term experimental platform was established in January 2023 at INIA Glencoe Experimental Station (Paysandú, Uruguay) as a long-term experiment designed to evaluate the effects of increasing grazing management intensity on native grassland-based livestock systems. The platform follows a farmlet-based approach, where each experimental unit represents a simplified but realistic livestock production system operating under contrasting grazing management strategies.

The experimental design consists of three contiguous but independent farmlets of 50 ha each, located on basaltic native grasslands with identical initial conditions of vegetation, soils, and topography characterized by field surveys and remote sensing. The conceptual framework and co-innovation process underpinning the design of this platform are described in detail in Devincenzi et al. (2021).

### Contrasting grazing management systems

**Figure 1.**
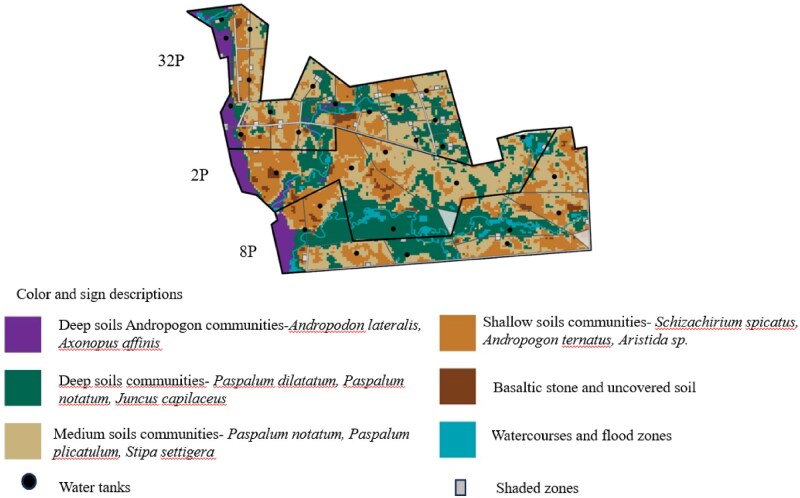
Experimental layout of the GLENCOE long-term experimental platform. Colors represent different plant communities. 2P: farmlet with grazing managed in 2 paddocks, 8P: farmlet with grazing managed in 8 paddocks, 32P: farmlet with grazing in 32 paddocks.

The three farmlets differ exclusively in the degree of spatial subdivision and associated temporal control of grazing, representing increasing levels of grazing management intensity (Figure 1):

- Two Paddocks (2P): a low-intensity system with two paddocks, representative of traditional extensive grazing with limited spatial and temporal control of forage utilization.
- Eight Paddocks (8P): an intermediate-intensity system with eight paddocks, enabling rotational grazing and moderate control of grazing timing and rest periods.
- Thirty-two Paddocks (32P): a high-intensity system with 32 paddocks, allowing fine spatial and temporal control of grazing, short occupation periods, and targeted rest and deferment of specific plant communities.

In all farmlets, paddocks were defined considering dominant plant communities to enable management decisions aligned with vegetation heterogeneity.

Importantly, all farmlets were established with an identical initial SR and managed under a shared decision-making framework, with differences restricted to the degree of paddock subdivision and monitoring frequency.

All the farmlets have similar proportions of the four plant communities, which were identified by topography and vegetation indexes (NDVI) provided by SENTINEL 2 (Level 1C; 10 m spatial resolution and <20% of cloud cover) from January 2017 to December 2019, and the 30 m spatial resolution from Digital Elevation Model DEM (SRTM) as described by Devincenzi et al. (2021). Plant communities at the beginning of the experiment were mainly composed of 1) *Schizachyrium spicatum*, *Andropogon ternatus*, and *Aristida echinulata*, 2) *Paspalum notatum*, *Bothriochloa laguroides*, and *Paspalum plicatulum*, 3) *Paspalum dilatatum, Juncus capillaceus*, and *Paspalum notatum*, and 4) *Andropogon lateralis* and *Axonopus affinis*.

### Livestock production systems and productive itineraries

**Figure 2.**
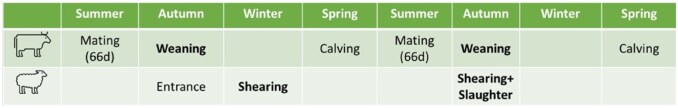
Animal productive itinerary of cow–calf operations and wool and lamb meat production of the GLENCOE long-term experimental platform.

Each farmlet simulates a mixed livestock system combining beef cow–calf production with sheep meat and ultrafine wool production (Figure 2). The cattle component is based on multiparous Hereford cows managed in a defined reproductive calendar.

The mating period extends for 66 d, typically from December to February, and calves are weaned at approximately 6 mo of age. Early weaning may be applied when technical recommendations are fulfilled (Rovira, 1996). Cow replacement decisions are based on fertility, health status, age, and temperament.

Sheep component involves growing and finishing lambs from weaning to slaughter, including two shearings, one in the first spring and a second in autumn, before slaughter. Mixed grazing between cattle and sheep is applied consistently across systems, maintaining a stable species ratio (2 lambs:1 cow) and allowing assessment of grazing complementarity under different management intensities.

### Adaptive management and decision-making framework

All animal and pasture management decisions implemented within the GLENCOE Platform are guided by an integrated decision-making framework that synthesizes the body of knowledge on native grassland management in Uruguay. This framework builds upon decades of experimental evidence, technical guidelines, and accumulated expertise developed by INIA and collaborating institutions. Management decisions are not applied unilaterally, but are discussed, agreed upon, and periodically reviewed through a formal co-learning process.

**Figure 3.**
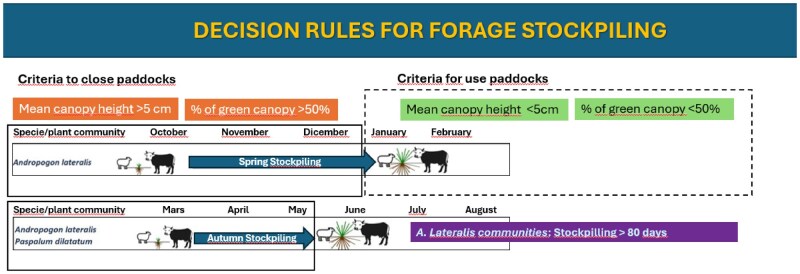
Decision rules for forage stockpiling for GLENCOE long-term experimental platform. Forage can be stockpiled depending on plant community and canopy height.

The criteria, indicators, and decision rules for animal and pasture management are documented in the operational protocol of the GLENCOE long-term experiment (Montossi et al., 2023). This protocol provides a shared reference framework that ensures consistency, traceability, and comparability of management actions across systems and over time, while allowing adaptive responses to climatic variability and system feedback. The protocol thus plays a central role in aligning scientific objectives with real-world management constraints and stakeholder expectations. A defining characteristic of the GLENCOE Platform is the implementation of adaptive management based on explicit decision rules. Key management decisions focus on forage stockpiling and SR adjustments and are defined separately for each system based on its specific characteristics. Forage stockpiling may be implemented during both spring and autumn, with its feasibility and duration determined by plant community composition, canopy height, and the proportion of green material in the standing biomass of each system (Figure 3).

**Figure 4.**
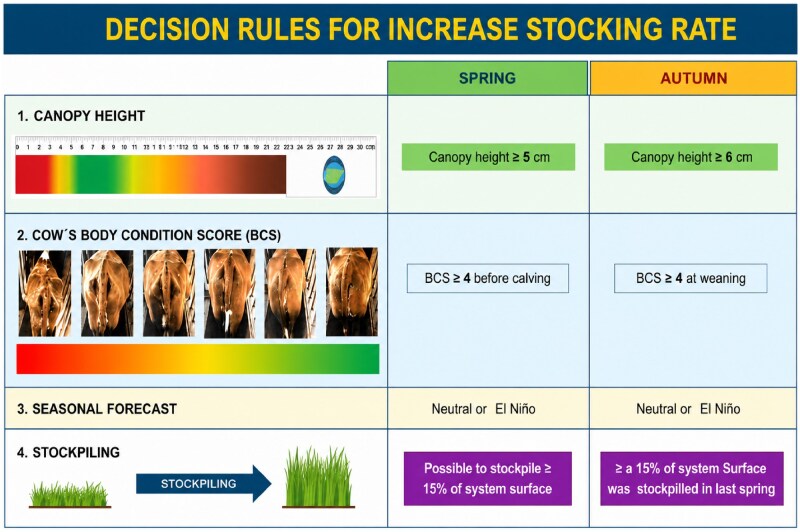
Decision rules for stocking rate adjustment in order to increase the number of animals in each system.

SR adjustments, in order to increase the number of animals in each system, are determined seasonally (spring or autumn). These decisions are based on canopy height, cow body condition score (BCS) (scale 1 to 8), seasonal weather forecasts, and the potential for forage stockpiling (Figure 4).

### Key variables and dimensions evaluated

System performance is assessed through a multidimensional framework capturing productive, environmental, economic, human, and emergent properties (Table 1).

**Table 1.** Main traits evaluated in each dimension on GLENCOE long-term experimental platform

Dimensions	Traits
Economic	Gross and net income Sensibility analysis
Environmental	Water quality Soil carbon Soil protein Gas emissions Diversity of plant species Ecosystem integrity index
Human	Working hours Work environment satisfaction
Emerging proprieties	Resilience facing droughts Stability of vegetal and animal production
Productivity	Forage production and quality Animal production (body weight, body condition score, fertility, wool production and quality, lamb meat quality)

Key dimensions include:

- Vegetation and pasture dynamics, including canopy height, forage availability, seasonal growth patterns, and plant community composition.
- Animal performance, encompassing body weight, BCS, reproductive performance, wool production and quality, and carcass traits in lambs.
- Environmental indicators, such as soil carbon stocks, soil biological activity, water quality, plant diversity, and an indicator of ecosystem integrity.
- Economic outcomes, including gross and net income and sensitivity analyses of system performance.
- Human and operational aspects, including labor requirements and working conditions.
- Emergent properties, particularly system stability and resilience to climatic variability, of production over time.

This integrated assessment framework facilitates the identification of trade-offs, synergies, and lagged responses that are fundamental to understanding the long-term consequences of grazing management intensification in native grasslands.

## The Role of the Support Group in Adaptive Management and Colearning

A distinctive feature of the GLENCOE Platform is the active engagement of a support group that accompanies the experiment from its initial design through ongoing implementation. This group comprises experienced livestock producers, extension agents, and researchers, selected for their leadership, practical expertise, and complementary knowledge of grazing systems. It serves as a formal space for dialogue, learning, and collective decision-making, bridging scientific evidence with real-world experience.

Beyond guiding adaptive management decisions, the support group plays a strategic role in shaping the platform’s direction. It helps define overarching objectives, prioritize research questions, and align experimental decisions with both scientific goals and producers’ needs. In doing so, the group ensures the platform remains relevant, forward-looking, and responsive to emerging challenges in native grassland-based systems.

Within the adaptive management framework, the group participates in regular evaluation workshops where system performance is assessed using shared indicators such as canopy height, animal body condition, and seasonal climate outlooks. Based on these integrated assessments, it contributes to decisions on SR adjustments, forage deferment strategies, and other tactical interventions. This process promotes transparency, evidence-based decision-making, and consistency with experimental objectives, while remaining grounded in commercial realities.

The group also plays a key role in implementing, transferring, and communicating knowledge generated by the platform. Leveraging members’ networks with producer organizations, extension services, and institutions, it facilitates the translation of experimental insights into technical messages, training activities, and communication products tailored to diverse audiences. This strengthens the feedback loop between research, practice, and extension, supporting the scaling of lessons learned beyond the platform’s boundaries.

Beyond its operational and strategic roles, the group critically examines underlying assumptions, identifies emerging issues, and contributes to the interpretation of system responses. Its involvement enhances the robustness of the experimental process by reducing reliance on individual judgments and promoting shared decision-making criteria. In this context, the support group operates as a collective cognitive and institutional framework that fosters learning at both the system and organizational levels.

In long-term grazing experiments, where outcomes depend on cumulative processes and delayed responses, governance and decision-making structures are as critical as experimental design. The support group embedded within the GLENCOE Platform reflects an explicit recognition that sustainable intensification of native grasslands requires not only ecological and technical innovation, but also social, institutional, and communicational arrangements that enable adaptive management and collective learning over time.

## Early System Trajectories during the First 3 Yr of the GLENCOE Platform

Given the long-term nature of the GLENCOE Platform, the present study focuses on early system trajectories observed during the first 3 yr of implementation. Rather than drawing definitive conclusions, this analysis provides initial insights into how contrasting grazing management intensities influence pasture structure, animal condition, and SR dynamics, setting the foundation for future evaluations of productivity, resilience, and ecosystem services over longer temporal horizons.

### Canopy height trajectories

**Figure 5.**
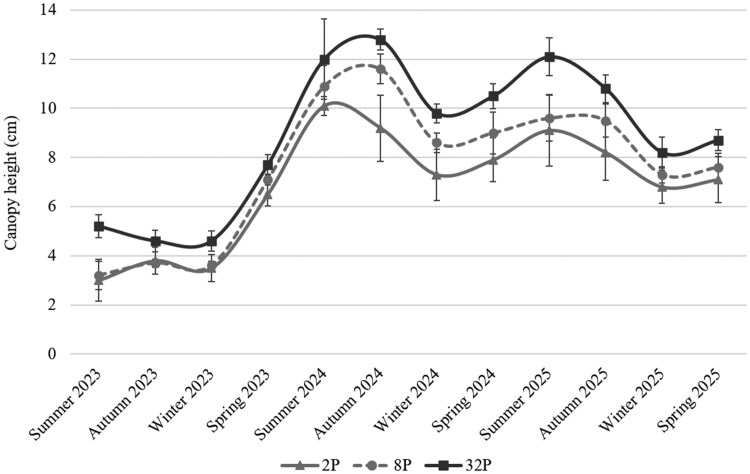
Seasonal variation in canopy height from 2023 to 2026 for the three grazing systems, showing generally greater canopy heights in the 32P system.

Throughout the 3 yr of evaluation, canopy height trajectories of the farmlets, especially from autumn 2024 to summer 2025, and from spring 2025 to summer 2026, revealed consistent statistics differences when 32P was compared with 2P (more than a standard error) among grazing systems, despite all farmlets being managed within a common minimum target range (4 to 6 cm) (Figure 5). In all farmlets, canopy height decreased from autumn to winter and then recovered in spring. This confirms that observed patterns reflect system-level responses to seasonal weather and not to grazing management.

Across all systems, canopy height followed a marked seasonal pattern, with higher values typically observed from spring to autumn, followed by declines during winter. However, the magnitude of these seasonal fluctuations and their consistency across years differed substantially among systems.

The high-intensity system (32P) showed greater canopy height than the other systems during summer 2023, primarily due to operational constraints associated with fence and water infrastructure, which limited grazing intensity prior to the onset of the experiment. These initial differences were resolved by spring 2023, after which 32P consistently exhibited higher average canopy heights across seasons and years, especially during periods of rapid forage growth. This pattern was evident in successive springs, where 32P showed a greater capacity to accumulate and retain standing biomass. Importantly, this advantage was not limited to peak growth periods, but extended into summer and autumn, suggesting a carry-over effect of improved forage production and structure resulting from finer spatial and temporal grazing control.

In contrast, the low-intensity system 2P displayed lower average canopy heights across most seasons. Seasonal troughs were deeper and more variable across years, indicating stronger coupling between forage growth conditions and grazing pressure. The intermediate system 8P exhibited canopy height trajectories that were consistently positioned between 2P and 32P, with seasonal dynamics reflecting a balance between forage capture and utilization. This system showed moderate capacity to sustain canopy height during less favorable seasons while avoiding the sharper fluctuations observed in 2P.

During spring, differences among systems were most pronounced, with 32P consistently reaching higher canopy heights due to its greater capacity to capture rapid forage growth, 8P showing intermediate accumulation, and 2P exhibiting lower heights associated with prolonged grazing pressure. In summer, canopy height continued increasing across all systems, but 32P maintained comparatively higher and more stable values, indicating buffering against climatic variability, whereas 2P experienced sharper declines and greater variability. Autumn patterns reflected contrasting canopy heights recovery capacities. In autumn 2024, there was a slight increase in 32P and a slight decrease in 2P compared to summer 2024; while in autumn 2025, all farmlets reduced the height of the forage. Winter canopy heights were lowest across systems, yet 32P entered this period with higher residual heights, while 2P was frequently below the canopy height threshold, reducing management flexibility and increasing vulnerability to overgrazing.

### SR adjustments

SR was expressed as livestock units per hectare (UG/ha), where one livestock unit (UG) corresponds to an adult cow of approximately 380 kg body weight under Uruguayan conditions. All farmlets started with the same initial SR and comparable proportions of the main plant communities (0.75 stock units/ha). SR is the primary driver of the evaluated traits, and its adjustment within each system is based on technical criteria established by a support group, which convenes twice annually: in autumn after calf weaning and in spring prior to the mating season. The technical parameters used for adjusting the SR are canopy height, animal BCS (on a 1 to 8 scale) during the latest evaluation period, and the climate prediction for the subsequent period.

Canopy height is monitored monthly, and the objective is to maintain the farmlets in a similar canopy height above a minimum a target range of 4 to 6 cm across all farmlets to ensure valid comparisons. BCSs are also monitored monthly, except during the calving period (September and October). BCS must be at least four points before calving and at weaning to allow SR increases.

Since the implementation of the GLENCOE Platform, three SR adjustments have been carried out—in autumn 2024, spring 2024, and autumn 2025—following the predefined decision rules and seasonal climate outlooks.

Autumn 2024. Mean canopy heights were 5.9, 6.3, and 7.2 cm for 2P, 8P, and 32P, respectively. Mean cow BCS at weaning was 4.8 for 2P and 8P and 5.1 for 32P. Climate forecasts indicated a higher probability of normal precipitation and temperature. Under this scenario, the SR was maintained in 2P, while increases of approximately 3% and 13% were implemented in 8P and 32P, respectively.

Spring 2024. Mean canopy heights increased to 7.9 cm in 2P, 9.0 cm in 8P, and 10.5 cm in 32P. Mean cow BCS before calving was 5.3 in 2P and 5.1 in both 8P and 32P. Climate forecasts indicated a higher probability of normal precipitation and temperature during spring–summer. Consequently, SR was maintained at 2P, while increments were implemented at 8P (addition of one heifer and two lambs) and 32P (addition of three heifers and six lambs).

Autumn 2025. Mean canopy heights were 8.0 cm in 2P, 9.3 cm in 8P, and 10.8 cm in 32P. Mean cow BCS was 5.0 in both 2P and 8P, and 4.7 in 32P. Climate forecasts indicated a higher probability of near-normal precipitation and temperature during autumn–winter 2025. Based on this scenario, the support group decided to maintain the SR in 2P and to increase it in 8P by adding three cows and six lambs. In 32P, the SR also increased by adding four cows and eight lambs.

Spring 2025. Mean canopy heights were 6.6, 7.3, and 8.4 cm in 2P, 8P, and 32P, respectively. Mean cow BCS before calving was 5.1 in 2P, 4.9 in 8P, and 4.5 in 32P. Climate forecasts indicated a greater probability of below-normal precipitation during November and December 2025. Considering this scenario, it was decided to maintain SRs unchanged across all systems during the spring–summer 2025 period.

**Figure 6.**
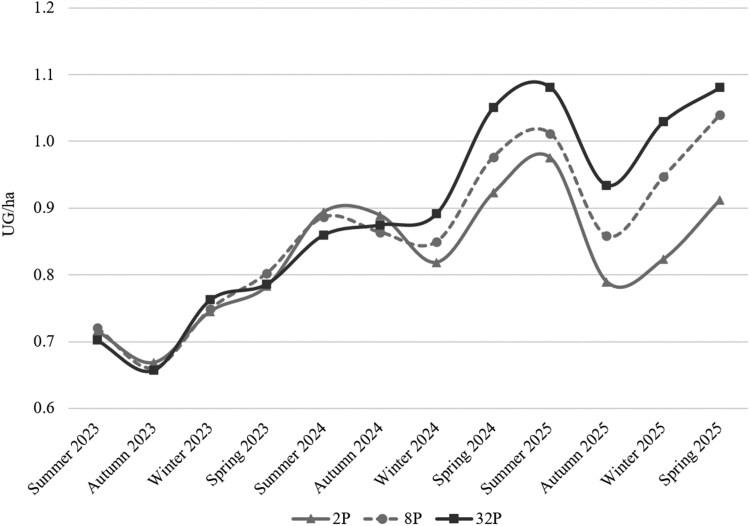
Seasonal evolution in stocking rate (UG/ha) from 2023 to 2025. 1UG=livestock unit- corresponds to an adult cow of approximately 380 kg body weight under Uruguayan conditions. Points represent the mean seasonal stocking rate. Seasons were defined as: Summer (January–March), Autumn (April–June), Winter (July–September), and Spring (November–December). Farmlets are indicated by symbols: 2P (triangles), 8P (circles), and 32P (squares).

**Figure 7.**
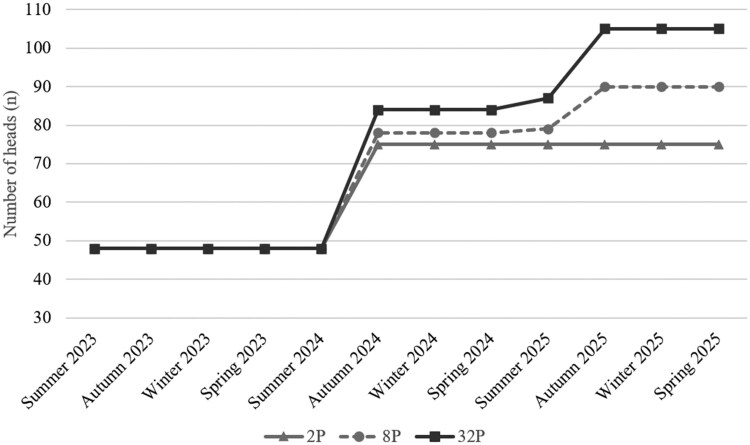
Seasonal changes in herd size (number of heads). From summer 2024 onward, a stocking ratio of two lambs per cow was applied across all farmlets. Farmlets are distinguished by symbols: 2P (triangles), 8P (circles), and 32P (squares).

SR and herd trends in each farmlet are shown in Figures 6 and 7.

### Support group evaluations and perceived impacts

The support group plays a key role in identifying system-level responses of native grasslands and livestock to the applied grazing management. Such responses require sustained observation over time for proper interpretation, thereby addressing a critical limitation of conventional short-term research. Participants emphasize the quality of the collective decision-making process fostered by the platform, where scientific evidence, field-based experience, and climatic context are jointly integrated (Porcile et al., 2025). This shared evaluation framework is perceived as reducing individual bias, strengthening confidence in management decisions, and improving understanding of system trade-offs and trajectories.

Additionally, the GLENCOE Platform is widely recognized as a space for co-learning, where continuous interaction among producers, technicians, and researchers enhances relevance, credibility, and potential adoption of the generated knowledge. Overall testimonies indicate that GLENCOE is perceived not only as a research experiment, but as a co-innovation that integrates science and practice to support the sustainable management of native grasslands under increasing uncertainty.

## Final Remarks

The GLENCOE Platform represents a methodological advance in a system-level experimental approach based on adaptive management decisions to evaluate the long-term trajectories of different grazing intensities. This design allows the evaluation of cumulative, delayed, and interactive effects of grazing management intensity under realistic production conditions, which are critical for understanding the functioning and sustainability of extensive livestock systems.

A key strength of the platform is the early and sustained involvement of end users—producers and extension agents—through the support group for co-innovation. Their participation from the conceptual stage onward has strengthened the relevance, credibility, and applicability of the experiment, fostering co-learning, shared decision-making, and more effective knowledge transfer.

This collaborative governance structure strengthens the platform’s potential to contribute to the sustainable management of livestock systems based on native grasslands of the Campos biome.

While the present paper focuses on early productive and structural trajectories, the coming years of the experiment will allow a comprehensive assessment of environmental indicators, including soil, biodiversity, and ecosystem integrity metrics. These future evaluations will be essential to fully quantify the environmental impacts of increasing grazing management intensity and to inform pathways for the sustainable intensification of extensive livestock systems under increasing climatic and socio-economic uncertainty.

From 2025 onward, this research platform became part of the Global Farm Platform, enabling the integration of long-term evidence from natural grassland–based livestock systems into globally harmonized sustainability metrics, including greenhouse gas emissions and efficiency indicators. This strategic integration strengthens the international relevance of extensive grazing systems, ensuring that science generated under native grassland conditions contributes directly to global assessments, modeling efforts and policy-relevant discussions on sustainable livestock production.

*Conflict of interest statement*. The authors declare no real or perceived conflicts of interest.

## About the Authors

**Fabio Montossi **graduated as an Agronomist from the University of the Republic (Uruguay) in 1989 and obtained his PhD from Massey University, New Zealand, in 1996. He currently serves as a Level I Researcher in Uruguay’s National System of Researchers (SNI, ANII). Since 1988, he has worked at the National Institute for Agricultural Research (INIA) of Uruguay, where he has held several leadership positions, including Head of the National Sheep and Goat Research Program, Director of the National Meat and Wool Research Program, Research Manager, and National Director (2015–2019). His research focuses on beef and sheep production systems, grazing management, animal nutrition, meat and wool quality, innovation, and technology transfer. He has extensive international experience in research collaboration, project evaluation, and agricultural development across Latin America, New Zealand, USA and Europe.

**Martín Jaurena** is a Principal Researcher in the Pastures and Forages Area of the National Institute for Agricultural Research (INIA), Uruguay. He obtained his PhD from the Federal University of Rio Grande do Sul (Brazil) and specializes in grassland ecology, grazing management, and natural grassland conservation. His research focuses on understanding plant–animal interactions, ecosystem services, forage production, and the sustainable intensification of grazing systems. Dr. Jaurena has led and participated in numerous national and international research projects and has contributed extensively to the scientific understanding, conservation, and sustainable management of South American native grasslands, particularly the Campos Biome.

**Thais Devincenzi** graduated from the Faculty of Agronomy at the Federal University of Rio Grande do Sul, Brazil, in 2008. She obtained her Ph.D. in Animal Science from the Federal University of Rio Grande do Sul in 2015. Between 2017 and 2019, she worked as a postdoctoral researcher at INRAE, France. Since 2019, she has been an Adjunct Researcher at the National Institute of Agricultural Research (INIA), Uruguay.Her research focuses on sustainable beef cattle production systems, including grazing management, reproductive and feed efficiency, enteric methane emissions, and climate resilience in grassland-based livestock systems. She is involved in multidisciplinary projects integrating animal, pasture, and environmental indicators to improve the sustainability and efficiency of beef production in South America. She also contributes to technology transfer, scientific training, and international research collaborations in livestock production systems.

**Virginia Porcile** is an Agricultural Engineer (MSc) and Extension Specialist at the National Institute for Agricultural Research (INIA), Uruguay. Her work focuses on sustainable livestock systems, family farming, technology transfer, co-innovation, and participatory approaches to rural extension and development. She has played a key role in the design, implementation, and evaluation of innovation initiatives involving livestock producers and other stakeholders, with particular emphasis on grazing-based beef and sheep production systems. She has extensive experience in facilitating collaboration among producers, researchers, extension services, and institutions to enhance the productivity, resilience, and sustainability of livestock systems in Uruguay and internationally.
